# The Effects of the Driver’s Mental State and Passenger Compartment Conditions on Driving Performance and Driving Stress

**DOI:** 10.3390/s20185274

**Published:** 2020-09-15

**Authors:** Víctor Corcoba Magaña, Wilhelm Daniel Scherz, Ralf Seepold, Natividad Martínez Madrid, Xabiel García Pañeda, Roberto Garcia

**Affiliations:** 1 Department of Computer Science, University of Oviedo, 33003 Oviedo, Spain; xabiel@uniovi.es (X.G.P.); garciaroberto@uniovi.es (R.G.); 2 Ubiquitous Computing Lab, Department of Computer Science, University of Technology, Business and Design Konstanz, 78462 Konstanz, Germany; wscherz@htwg-konstanz.de (W.D.S.); ralf@ieee.org (R.S.); 3 Institute of Digital Medicine, I.M. Sechenov First Moscow State Medical University, 119435 Moscow, Russia; nati@ieee.org; 4 IoT Lab, School of Informatics, Reutlingen University, 72762 Reutlingen, Germany

**Keywords:** driving safety, driving emotions, driving stress, lifestyle, sensors, heart rate

## Abstract

Globalization has increased the number of road trips and vehicles. The result has been an intensification of traffic accidents, which are becoming one of the most important causes of death worldwide. Traffic accidents are often due to human error, the probability of which increases when the cognitive ability of the driver decreases. Cognitive capacity is closely related to the driver’s mental state, as well as other external factors such as the CO_2_ concentration inside the vehicle. The objective of this work is to analyze how these elements affect driving. We have conducted an experiment with 50 drivers who have driven for 25 min using a driving simulator. These drivers completed a survey at the start and end of the experiment to obtain information about their mental state. In addition, during the test, their stress level was monitored using biometric sensors and the state of the environment (temperature, humidity and CO_2_ level) was recorded. The results of the experiment show that the initial level of stress and tiredness of the driver can have a strong impact on stress, driving behavior and fatigue produced by the driving test. Other elements such as sadness and the conditions of the interior of the vehicle also cause impaired driving and affect compliance with traffic regulations.

## 1. Introduction

In 2018, there were 102,299 traffic accidents with victims in Spain, with 1806 people losing their lives [1]. Most of these accidents happened in cities. Distractions were the main cause of fatal accidents at 32%, speed was at 22% and alcohol or drug consumption was at 21%. Most accidents are due to human error [2,3]. Other factors such as the environment or the vehicle involve less hazard [4].

In the literature, we find many works that evaluate driving performance and the drivers’ physiological and cognitive states [5,6]. For example, in [5], the authors proposed a method to determine a driver’s relative stress level based on analyzing physiological data and artificial intelligence. Twenty-four drivers participated in the experiment. The authors monitored the participants in real driving for at least 50 min. The results showed that the conductivity of the skin and the pulse metrics are those that most correlate with the level of stress. In [6], the changes in the muscles of the shoulder and the neck were analyzed while the participant drove in a driving simulator. Professional and non-professional drivers participated in the study. The conclusions were that, in both cases, the drivers suffered from fatigue after driving for 15 min. Many researchers have found a strong relationship between emotions and traffic accidents. Researchers in [7] conducted a cluster analysis on responses to a survey by more than 1500 college students about potentially annoying driving-related situations. The authors noted that men were more angered by the police presence and slow driving. On the other hand, women were more angered by illegal behavior and obstructions. Finally, they concluded that knowing the driver’s anger could be of great help in reducing traffic accidents. Negative emotions, such as fear, anger, disgust or sadness, increase the probability of manifesting dangerous behaviors while driving. In [8], the authors analyzed the influence of mood on risk perception and attitude. The authors conducted an experiment where they induced different emotions in the driver by watching videos. The results showed that negative emotions significantly increase the perception of risk by the driver, but also cause an inappropriate attitude. In [9], the authors focused on studying how sadness affects driving performance. Sixteen drivers participated in the experiment. They used a driving simulator. The results showed that drivers with sadness make more driving mistakes than neutral drivers. Furthermore, the driving times is also longer. In [10], the authors explore the use of affective interfaces in vehicles. The researchers conducted an online survey on emotional situations on the road. The results showed that drivers who experienced negative situations require better information management and a high degree of automation. In contrast, drivers with positive emotions prefer a more genuine driving mode.

The state of the interior of the vehicle is another factor to consider. Other aspects such as the number of occupants in the vehicle or adequate ventilation should also be taken into account. It has been shown that a high concentration of CO_2_ decreases cognitive ability and increases fatigue. In [11], the authors analyzed the performance in a cognitive test when the participants were exposed to different levels of CO_2_. Participants obtained significantly better cognitive scores when the CO_2_ level was lower than 1400 ppm. Similar results were obtained in [12]. In this work, the researchers analyzed the effects of CO_2_ on decision making. Twenty-two people participated in the experiment who took a decision-making test. In addition, the participants also filled out a questionnaire about their health and perceived air quality. The authors found that there was a moderate worsening of decision-making performance at 1000 ppm of CO_2_. 

Autonomous vehicles could be a solution to reduce traffic accidents caused by human errors. However, on the one hand, these solutions still have a high cost and are not accurate enough [13]. In addition, in most countries, there are still no laws or regulations for this type of vehicle [14,15]. On the other hand, the autonomous vehicles that have been developed to date require that the driver maintain attention on the road and if an exceptional event takes place, he or she must take control. The first accidents have already occurred due to a lack of attention and the need for traditional and autonomous vehicles to coexist. In [16], the author used a Tesla Model S vehicle for six months. In this article, he analyzed the problems that these vehicles present for the driver in real driving conditions. The researcher points out that these vehicles can improve safety and driving comfort, but also can present challenges due to the transition between automatic and manual driving mode. Finally, a series of guidelines are proposed to improve the way in which this type of vehicle interacts with the driver. Reducing human errors while driving remains a significant concern.

The emotions experienced by the driver can be caused by events that occur during the driving task or before. In [17], McMurray analyzed driving reports. The researcher concluded that drivers who were in the process of a divorce had a higher probability of suffering traffic accidents and violating traffic rules than other drivers. Similar results were obtained by [18], where the authors examined how stressful environments and psychological characteristics of the driver affect driving behavior. The results showed that stress significantly increases the risk of traffic accidents. The researchers also highlighted the importance of experiential knowledge acquired without instruction to present good driving behavior. In [19], the authors found that financial problems increase the likelihood of suffering a traffic accident. Therefore, events unrelated to the driving task influence stress and driving skills.

Many of the works that analyze the driver’s mental state and its effect on driving are based on artificially inducing a certain emotion using music, images or words [20]. For example, in [21], the researchers studied the effect of joy, sadness and anger on driving behavior using induced emotions. The results showed that negative emotions cause dangerous driving behaviors. The authors also observed that, in some cases, the emotional state did not affect driving when the workload was high. In [22], the authors conducted an experiment where participants were induced with sadness, anger or neutral emotions. Participants with induced anger or sadness made more driving mistakes than participants induced with neutral emotions.

Originally, the works that analyzed drivers’ emotions used subjective measures [7]. These methods require the direct intervention of the participant. Therefore, the samples are obtained at a low rate. In recent years, the psychological methods have become less expensive and intrusive due to wearables. These devices allow us to monitor the driver continuously and without requiring his direct involvement. [23]. During driving, the use of non-intrusive devices is essential so as not to affect driving performance or cause safety problems [24,25]. In [26], we can find a review of the solutions to monitor a driver’s psychological state.

### Contribution

Most of the works documented in the literature focus on ascertaining the emotions of the driver while driving. There are some studies in which the driver’s previous state is analyzed, but they are minimal. Furthermore, they do not investigate how emotions together with factors such as the interior state of the vehicle (temperature, humidity and CO_2_ concentration) or the music the driver listens to affect driving and the level of stress. Another problem we have found in previous works is that they artificially induced moods, which could lead to inaccuracies in the results.

The objective of this work is to analyze which elements affect driving behavior and stress levels, focusing on the drivers’ initial emotions, their characteristics and the comfort inside the car. The conclusions of this analysis can be used to include the emotional component in driving assistants. Most of them are limited to warning the driver when they invade the opposite lane or exceed the speed limit.

## 2. Related Work on Stress Detection

Stress is defined as a state of physical, psychological or emotional health experienced by a person when the perceived or actual demand requires a high number of resources [27]. Stress appears when the demand for mental workload exceeds the capabilities of the subject [28]. Stress may be accompanied by other emotions such as anxiety [29]. However, it is not always bad. Healey et al. [5] classified stress into two types: eustress and distress. Eustress encourages people to achieve a high level of performance. If the level of stress is too low, it can cause drivers to suffer fatigue and drowsiness, and they may lose control of the vehicle [30]. Distress appears when there is an excess in the level of demand that surpasses the capacity of the person and consequently discourages the driver [31]. The level of stress experienced while driving can be affected by four factors: the physical and mental condition of the driver, road and traffic conditions, vehicle condition and external disturbances. This paper focuses on the driver’s mental state and vehicle condition (music tempo and CO_2_ concentration).

Stress detection methods can be classified into four categories:

- Self-report questionnaire assessment;
- Physiological measures;
- Driving behavior monitoring;
- Visual-based and speech detection.

The self-report questionnaires analyze the driver’s behavior and strategies for coping with different types of stressful events. In addition, the characteristics of the driver are very important [32]. Data such as age or accident history have a strong relationship with stress. Drivers who have suffered more traffic accidents are more likely to feel anxiety and develop post-traumatic stress disorders [33]. One of the most used questionnaires in research is the driver behavior inventory (DBI) [34]. In this questionnaire, stress is defined by five elements: driving aggression, dislike of driving, tension and frustration connected with successful or unsuccessful overtaking, irritation when overtaken and heightened alertness and concentration. There are also many other questionnaires such as: the driving stress inventory (DSI) [35], stress arousal checklist (SACL) [36] and Dundee stress state questionnaire (DSSQ) [37]. In the case of workload measurement, NASA load index (NASA-TLX) [38] and driving activity load index (DALI) [39] are the most widely used. In the experiments, several of them can be used with different objectives. For example, in [40], the participants completed the DSI questionnaire before the test to estimate their vulnerability to stress. They then completed the DSSQ questionnaire to analyze the stress and workload caused by the task.

Stress detection models based on physiological signals allow us to objectively monitor the driver’s stress level in real time. They mainly use the heart rate signal, skin conductance, skin temperature and the encephalogram [41,42]. The main disadvantage of these methods is that they require the use of sensors, which increases the cost and reduces the number of potential participants. In addition, these solutions can cause discomfort if they are intrusive. However, in recent years, wearable devices have been developed that can monitor the driver without affecting mobility and at a relatively low cost. An example widely used in research is the Empatica E4 [43]. These portable devices are not as accurate as medical devices. However, there is a strong correlation between them, and they are valid for measuring stress and conducting long-term studies [44,45].

In the literature, we find many proposals of stress detection based on these types of signals. A strong relationship between driving stress and heart rate and blood pressure was reported in [32,46]. In [47], the authors proposed a binary logistic regression model to predict driving stress. This method uses galvanic skin response data obtained in real road driving situations to predict whether driver stress will be high or low. GSR data were collected using a wearable device (Empatica E4). The main advantage of this solution is that it is non-intrusive so it can be used in real driving. The authors achieved an accuracy higher than 80% using this model. In [48], the authors wanted to analyze the relationships between driving stress, traffic conditions and road types. The authors proposed using electrodermal activity (EDA) signals to estimate the levels of driving stress taking into account the road type and traffic conditions. The classification model developed was based on the data collected by a driver in real road driving conditions for 60 min a day for 21 days. The results showed than traffic conditions and road type are factors that influence driving stress.

Proposals using physiological signals can detect driver stress in real time using artificial intelligence algorithms [5,49]. Galvanic skin response (GSR) and heart rate variability (HRV) are considered the best indicators of stress in real time [5]. However, we should take into account the latency that in the case of skin conductivity can be up to 1.4 s [50].

A different alternative to using these sensors is to analyze the driver’s face and speech. This avoids having to wear sensors. For example, the authors in [51] used visual-based thermography to detect facial skin temperature. In [52], the authors proposed to analyze facial expression using an NIR camera. The drawback is that good illumination is required to achieve accuracy in stress detection. Voice speech is another variable that can be helpful for detecting stress. In [53], the authors analyzed the changes in pitch of the subjects to detect stress. The problem with this type of approach is that it requires the driver to perform additional tasks while driving in order to make the voice recording, which could cause distractions [54]. In addition, noise inside the vehicle cabin could make it difficult to detect stress [55].

There are some proposals to detect stress based on driving behavior. The authors in [56] highlighted that the autonomic system (ANS) and driving style change when the level of stress is high. Stressful events can be detected by analyzing the corrections the driver makes with the steering wheel and the pedals of the vehicle. In [57], the researchers proposed a system that monitors the turning patterns of the steering wheel and recognizes lanes and accelerating patterns in order to detect stress.

Finally, there are proposals that combine the use of physiological signals with vehicle telemetry (steering wheel movement, acceleration, deceleration). In [58], the authors presented a wearable glove system for monitoring stress while driving. The proposal extracted features of photoplethysmography (PPG) and inertial measurement unit (IMU) sensors located in the glove to assess the stress events. The proposal was able to detect stress events with an accuracy rate of over 95% using an SFS-SVM classifier with the RBF kernel function. The main limitation of this device is that participants cannot change the position of their hands on the steering wheel during the driving test.

## 3. Materials and Methods

**Figure 1:**
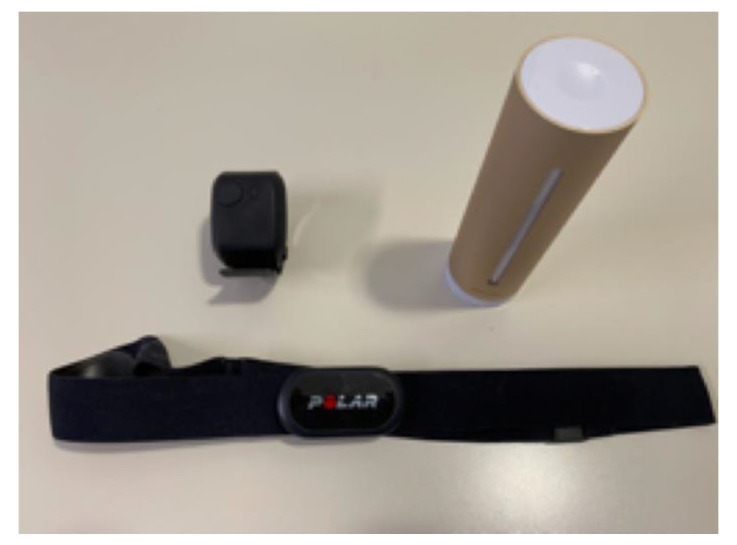
Sensors used in the experiment.

In this section, we will describe the materials and the procedure to carry out the experiment. We present the sensors (Figure 1) used to monitor driving stress, to evaluate driving performance and to obtain the state of the simulation environment (temperature, humidity and CO_2_ level). Driving stress is tracked using an Empatica E4 wristband and a Polar H10 chest band. The environment is supervised using the Netatmo device. We also define the measurements that we will use to evaluate the drivers and their driving behavior from the data gathered by the sensors. In addition, the test scenario will be detailed, explaining the simulator used, as well as the music that the driver listened to during the driving task. It will specify the survey completed by the drivers before and after the test to ascertain their characteristics, their opinion of the experiment and their physical and mental state.

### 3.1. Heart Signal

We have used several sensors in this work to measure stress objectively. One of the vital signs most used in research on driving is the heart rate variability (HRV). There are numerous studies where this biosignal is measured due to its strong correlation with stress, and the fact that it can be obtained in a non-intrusive way [5].

Heart rate variability can be analyzed in two different domains: time and frequency. Time domain analysis of the HRV signal consists of measuring the mean or standard deviation of the time intervals between consecutive heartbeats. Frequency domain analysis is a method based on the amount of heart signal found in two different frequency bands. In the case of heart rate analysis, the ranges are (0.04–0.15 Hz) and (0.15–0.4 Hz). In this work, we use the following measurements obtained from the heart rate signal that have been widely used in the literature [59]:

- pNN50 (%): this is the number of consecutive heartbeats differing more than 50 ms divided by the total number of measured heartbeats and expressed as a percentage. This variable decreases when driving stress is high.
- LF/HF: this is the low-frequency (LF) power (0.04–0.15 Hz) modulated by the sympathetic and parasympathetic nervous system divided by the high-frequency (HF) power (0.15–0.4 Hz) associated with the parasympathetic nerve activity. This ratio captures the global sympathovagal balance [25]. A high LF/HF ratio means sympathetic dominance, which happens when driving stress is elevated.

There are several non-intrusive devices which obtain the heart rate. Many of them are based on the photo-plethysmography sensor that allows us to measure the blood volume pulse. This type of device has improved significantly in recent years. However, they are very sensitive to movement and the level of pressure on the skin [60]. Another type of sensor that allows measuring the heart rate non-intrusively is a chest band with electrodes. These solutions achieve higher accuracy than the photo-plethysmography sensor [61,62].

In our case, the heart signal is obtained using a Polar H10 chest band. Polar H10 is the successor to the Polar H7 device. Polar H10 introduces improvements to measure heart rate variability. This device can offer precision for measuring the time between successive heartbeats (also called RR interval) similar to that obtained by a Holter ECG [61]. Polar H10 was connected wirelessly to the EliteHRV^©^ app [63] running on a Google Pixel 3a. This app directly receives the RR intervals and uses a proprietary algorithm to correct artifacts such as ectopic beats or signal noise to present a more valid signal for heart rate analysis (HRV). We exported the calculated HRV values (pNN50 and LF/HF ratio) from the web dashboard provided by this app. It is also important to mention that before starting the heart rate measurement, there is a 30 s sensor stabilization period. Table 1 shows the Polar H10 specifications.

### 3.2. Skin Conductivity

The variation of skin conductivity is linked to the sympathetic nervous system [62]. When the driver has high stress, the activity of sweat glands is triggered by postganglionic sudomotor fibers. The result is a change in the skin conductivity response (SCR) that can be measured by applying a low constant voltage. The SCR amplitude can be used as an indicator of sympathetic activity [33].

Skin conductivity is monitored using an Empatica E4 wristband. Table 2 [65] shows the characteristics of the sensors that the Empatica E4 device integrates. This device is certified as CE Medical class 2a [66] and has been validated in many works [67,68]. It includes a photo-plethysmography sensor that allows us to measure the blood volume pulse. It also has a galvanic sensor to measure sympathetic nervous system arousal as well as to derive features related to stress, engagement and excitement. The wristband features are a 3-axis accelerometer to capture motion-based activity and an infrared ray which reads peripheral skin temperature. This device has been designed for continuous, real-time data acquisition. 

However, the calculation of the amplitude is not trivial. Usually, the SCRs overlap each other. In the standard peak detection method (trough-to-peak), the SCR amplitude is obtained by calculating the difference between the peak and the previous trough of the skin conductance data. This results in an underestimation of the amplitude of subsequent SCRs. The degree of underestimation depends on the amplitude and proximity of the preceding SCRs. There are different proposals in the literature to avoid this problem. In this paper, a deconvolution approach [35] is used, which separates skin conductivity data into continuous signals of tonic and phasic activity. This algorithm allows us to represent the overlapping SCRs by compact impulses, thus avoiding the underestimation problem. To that end, we use Ledalab 3.4.9 [69], which is recommended by Empatica. Before the signal deconvolution by continuous decomposition analysis, we pre-process it to eliminate high-frequency noise by applying a smoothening filter consisting of a 4-sample Gaussian window.

### 3.3. Environments

Temperature, humidity and CO_2_ concentration are variables that influence comfort and safety [70]. Vehicles tend to circulate in areas that are heavily contaminated. Many drivers close the windows and use the air conditioning in order to avoid polluting gases. However, the air that comes from this system is not clean. Besides, the reduced space of the vehicle causes a high amount of CO_2_ to accumulate due to the passengers themselves. If the level of CO2 inside the vehicle is very high, the driver may suffer from dizziness and nausea [71].

Temperature and humidity are other factors that can induce fatigue in the driver [72]. In the past, temperature was a significant cause of traffic accidents [73]. Currently, most vehicles integrate an air conditioning system. However, it is very difficult to adjust it correctly because the thermal sensation is different for each passenger [74]. An inadequate temperature, either too high or too low, causes a significant worsening of driving performance [75]. In order to monitor the interior of the vehicle, we used a Netatmo Healthy Home Coach [76]. This system allows us to obtain the air temperature, relative humidity and CO_2_ concentration. The measurements are taken every five minutes, and are uploaded to the cloud instantly. The data are processed internally using proprietary Netatmo algorithms. We directly downloaded the temperature, humidity and CO_2_ values using a Python script. We could not obtain the raw data. Regarding the validity of the use of the device to measure the CO_2_ concentration, there are several works where it has been verified, providing that a calibration has been previously performed [77,78].

The Home Coach Netatmo device allows manual calibration of the CO_2_ and temperature sensor. The calibration of the CO_2_ sensor of the device was carried out for 8 h inside a room without any occupants following the manufacturer’s instructions [79]. The temperature of the room during calibration was 25 °C. The temperature sensor was calibrated by calculating the average difference between the measurement obtained by the Netatmo device and the value offered by a weather station belonging to the State Meteorological Agency (AEMET), located in Asturias. The samples were collected over 7 days at 11 A.M. As a result, we fitted the temperature by +0.1 °C. In order to obtain the temperature reading using the Netatmo device at the location of the weather station, an external Samsung power bank was used. The Netatmo device has been validated by other authors previously obtaining good accuracy when the value of the air temperature under investigation is close to the air temperature in which the manual calibration occurs. In our case, the temperature during the calibration and test drive was very similar. Table 3 shows the specifications of the air quality sensor.

In our experiment, the temperature and humidity remained constant and we analyzed the CO_2_ concentration. In the literature, we find works where the CO_2_ concentration inside the vehicle cabin is analyzed [80]. However, we have not found works which study how a high CO_2_ concentration influences driving stress and driving behavior.

### 3.4. Driving Simulator

This experiment was carried out using the “City Car Driving” simulator [82]. The simulator uses advanced car physics to achieve a realistic car feeling and a high-quality render engine for graphical realism. The simulator implements German traffic rules and warns drivers if they fail to comply with some of these. Traffic density can also be adjusted with the simulator. The drivers’ behavior and pedestrians’ behavior are sometimes erratic as in a real environment. The vehicles can collide with the player’s car or with each other. Pedestrians sometimes cross the road in the wrong places. The scene selected for the experiment is named “Old District” and is characterized by narrow streets with simple crossing places and clear traffic patterns. This driving simulator was developed to train novice drivers in driving schools. It saves a log file with all the traffic rules that the driver violated as well as events such as traffic accidents.

The execution of the driving simulator on a computer and features are included in Table 4. Three 27-inch screens were connected to the computer. To operate the vehicle, we employed a Logitech G29 [83]. This device is an electronic steering wheel designed for driving video games with realistic force feedback. It includes a set of three pedals and a gearbox, and it allows us to archive an immersive perception in the virtual environment. Table 5 shows the specifications of the device.

In order to evaluate driving performance, we have developed a program based on the SFML library [85] that captures the angle of rotation of the steering wheel and the pressure applied by the participant on the pedals. In this study, we have defined the following variables to assess driving behavior:

- Harsh braking: this is the percentage of time that the driver stopped abruptly concerning the total braking time. We have considered that the driver brakes sharply when the deceleration is −2.5 m/s^2^ or more. This value is considered by many authors as abrupt [86].
- Braking time: this is the time of the total driving time (25 min) that the driver was pressing the brake pedal and is expressed as a percentage.
- Harsh acceleration: this is the percentage of time that the driver sped up abruptly with respect to the total acceleration time. We have considered that the driver accelerates sharply when the value is 1.5 m/s^2^ or more. This value is considered by many authors as abrupt [86].
- Acceleration time: this is the time of the total driving time (25 min) that the driver was pressing the accelerator pedal and is expressed as a percentage.

### 3.5. Music Tempo

Many people listen to music when they are driving. Studies show that music influences human behavior. In supermarkets, fast music causes customers to move faster through the store [87]. In bars, fast music makes people consume their drinks quickly [88]. The music tempo also causes an effect on the speed and accuracy of the tasks. In [89], fast music increased the rate and accuracy of mathematical computations in stock market environments. In driving, fast music also instigates the driver to drive faster [90]. However, in music, many parameters can affect the driver, such as the genre of music, instruments or volume, but the tempo is one of the most important in driving.

In the experiment, the participants listened to music through headphones. Drivers could adjust the volume according to their preferences to avoid discomfort. We created two playlists on Spotify [91]. One includes music with a slow tempo (65–71 bpm), and the other contains audio tracks with a fast tempo (155–188 bpm). Each of the participants was randomly assigned one of the two lists. Further, the song “Sonata for Two Pianos in D major” from Mozart was used to relax the participant at the beginning of the experiment. All songs were reproduced with the best sound quality that Spotify allows (OGG, 320 Kbps). In addition to the music, the drivers listened through headphones to the sound of the vehicle’s engine.

### 3.6. Survey

The participants completed two surveys: one at the beginning of the experiment and another at the end. The purpose of the pre-test survey was to obtain driver characteristics and the emotional and physical states. The survey contains questions about the level of stress, fatigue and sadness that the driver feels before the driving test. The participant should respond using a Likert scale. A Likert scale is a psychometric scale used in educational and social sciences research that employs questionnaires [92]. The Likert scale is composed of a set of statements (items). Participants are asked to show their level of agreement (from strongly disagree to strongly agree) with the given statement (items) on a metric scale. In our case, the scale is between 1 and 5, where 1 means that he or she does not suffer from that symptom or emotion and 5 that he or she develops it to a high degree.

The post-test survey was focused on ascertaining the emotional and physical states after completing the driving task. The objective was to check if the driving task had emotionally affected the driver. As previously mentioned, it comprises queries about the level of stress, fatigue and sadness that the driver feels but in this case after the driving test. This survey also includes questions about the degree of realism of the simulator, the satisfaction level of the drivers with their driving performance and the environmental conditions (temperature, noise and humidity). These questions will allow us to check if the subjective opinion of the participant corresponds to the data gathered by the sensors and if the simulator is realistic enough to infer that in a real environment, we would obtain similar results.

### 3.7. Procedure Description

First, the sensors were fitted to the participant, who then completed the initial survey. Then, he or she listened to Mozart’s Sonata for Two Pianos in D major using headphones. This track has been selected because it improves mental function [93]. The objective of this phase is to be relaxed before the driving test and to stabilize the sensors. A total of 50 drivers with an average age of 31.76 years (max: 57, min: 18; std. dev.: 10.48) and driving experience of 11.28 years (max 40, min: 1, std. dev: 10.24) participated in the experiment. The participants drove for 25 min. In the driving test, the heart signal, the skin conductivity and the environment (temperature, humidity and CO_2_ level) were monitored. The music and the sounds of the vehicle were listened to through headphones. The drivers had to complete the routes proposed by the GPS of the driving simulator. Each route has a length of 5 km and its level of difficulty is comparable because the concentration of vehicles and pedestrians is the same in all cases. The driving simulator assigns points to the participant at the beginning of the route. Each time an infraction is committed, points are deducted. When the score is zero, the route must be repeated. This allows the participant to be focused on the driving task as if in a real environment [94]. The driving time is 25 min to have enough time for the stress data to be valid [95].

A statistical analysis was conducted using R (version 3.6.0) in order to obtain conclusions from the data. We have used the Student’s test or Wilcoxon’s test for independent samples depending on whether the hypothesis of normality is verified or not. The significance level was set at 0.05. Therefore, if the *p* value is less than 0.05, we assume that there are significant differences between the analyzed groups.

## 4. Results

### 4.1. Effects of Initial Stress

The drivers have been grouped into two sets, “stressed” and “non-stressed”, according to the initial level of stress. The drivers indicated their stress level using a Likert scale with values between 1 and 5, where 1 means that they are not suffering from stress and 5 that they have a lot of stress. The “stressed” group is made up of 21 drivers. These indicated in the initial survey that their stress level was equal to or higher than 4. The “non-stressed” group consists of 29 drivers. These drivers indicated a stress level equal to or less than 3. In order to analyze if there are significant differences between the two groups, we conducted a Student’s test or a Wilcoxon’s test for independent samples, depending on whether or not the normality hypothesis is verified. We use *p* < 0.05 as the significance level.

Table 6 shows the variables related to stress during driving. The participants who initially indicated that they had stress also obtained values associated with high stress during the driving test. We have found significant differences in two of the three variables analyzed. The result of Wilcoxon´s test is Z = −3.116, *p* < 0.05 for pNN50, Z = −3.803, *p* < 0.05 for LF/HF ratio and Z = −3.491, *p* < 0.05 for SCR amplitude.

Stress also has consequences on driving behavior. Stressed drivers accelerate and brake more frequently and intensively than other drivers, as can be seen in Table 7. The difference in driving behavior is especially important in harsh accelerations and decelerations. The percentage of sudden accelerations is six times higher compared to unstressed drivers and twice as high in the case of sudden braking. The result of Wilcoxon´s test is Z = −5.376, *p* < 0.05 for harsh braking, Z = −2.428, *p* < 0.05 for braking time and Z = −5.063, *p* < 0.05 for harsh acceleration. In the case of the acceleration time, neither the normality hypothesis nor the equality hypothesis of variances are rejected. Therefore, we carry out a Student’s test whose result is t(48) = 2.703, *p* < 0.05.

**Figure 2:**
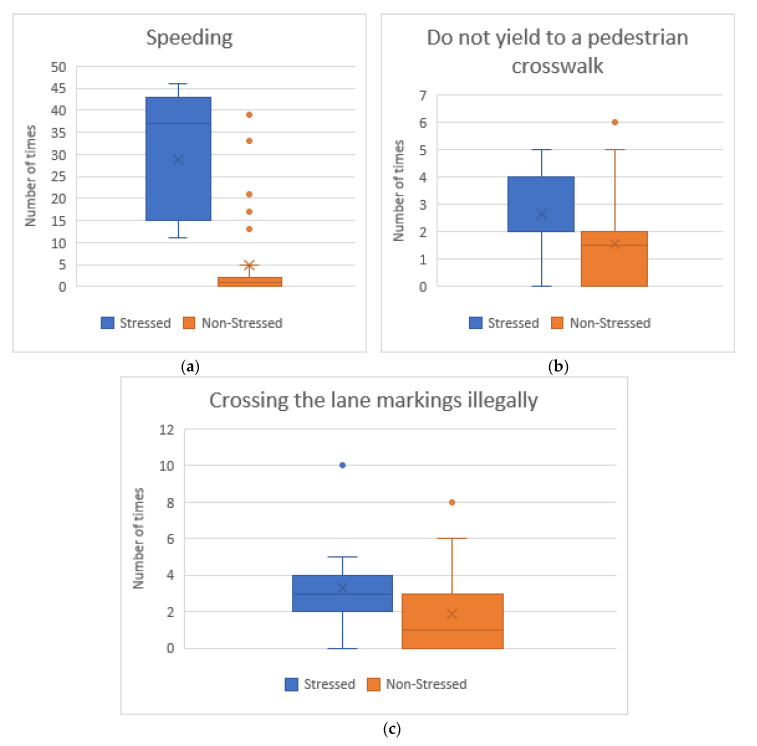
Average number of traffic rules broken grouped by initial stress level: (**a**) Speeding; (**b**) Do not yield to a pedestrian at a crosswalk; and (**c**) Crossing the lane markings illegally.

Figure 2 shows the degree of compliance with traffic rules grouped by initial stress level. We have found significant differences between stressed drivers and non-stressed drivers in “Speed limit exceeded” (Z = −5.184, *p* < 0.05), “Do not yield to a pedestrian in a crosswalk” (Z = −2.695, *p* < 0.05) and “Crossing the lane markings illegally” (Z = −2.588, *p* <0.05). Drivers who are initially stressed often drive at high speed, invade the opposite lane to overtake other vehicles and do not stop at crosswalks.

**Figure 3:**
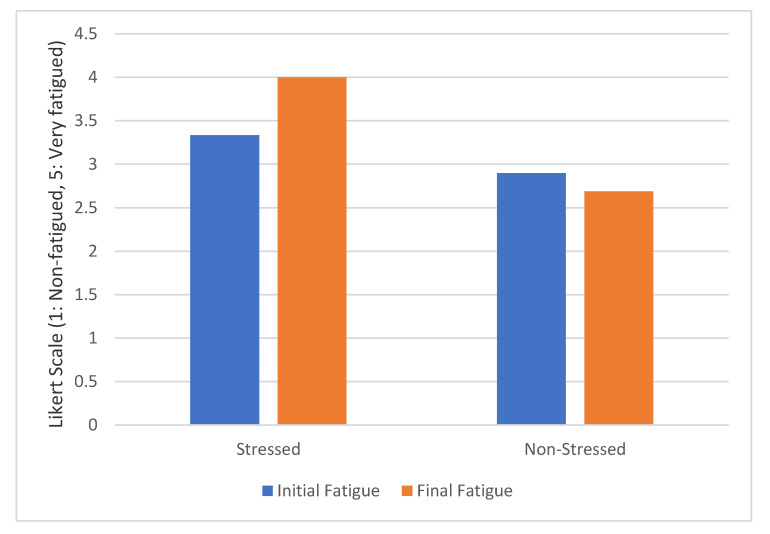
Fatigue evolution grouped by initial stress level.

Figure 3 compares the difference between initial and final fatigue for the two groups of drivers. These values were obtained from the pre-test and post-test surveys. On the one hand, stressed drivers suffer an important increase in the fatigue level after completing the driving test. The tiredness grew by 20%. In the case of drivers with low initial stress, the level of tiredness scarcely changed. On the other hand, at the beginning of the driving experiment, we found no significant differences in the fatigue level between the two groups of drivers analyzed. The result of Wilcoxon´s test is Z = −1.491, *p* > 0.05. However, we observed significant differences at the end of the experiment. The result of Wilcoxon´s test is Z = −4.545, *p* < 0.05.

### 4.2. Effects of Sadness

The drivers have been grouped into two sets according to the sadness level. The drivers indicated their sadness level using a Likert scale with values between 1 and 5, where 1 means that they are very happy and 5 indicates that they are very sad. The group of drivers with sadness is composed of 17 drivers. These indicated in the initial survey that their sadness level was equal to or higher than 4. The non-sadness group is formed by 33 drivers who rated their level of unhappiness with a value equal to or less than 3.

Table 8 shows the variables related to stress during driving. Drivers who show sadness are also those who have a higher level of stress. However, the differences are not significant. The result of Wilcoxon´s test is Z = −0.881, *p* > 0.05 for pNN50, Z = −0,522, *p* > 0.05 for LF/HF and Z = −0.420, *p* > 0.05 for SCR amplitude.

Table 9 presents the acceleration and deceleration values obtained by the drivers. We observe that drivers with sadness accelerate sharply more times than drivers without sadness, although the difference is not significant. The result of the Student’s test is t(48) = 2.001, *p* > 0.05. No significant differences were found either in the rest of the parameters.

**Figure 4:**
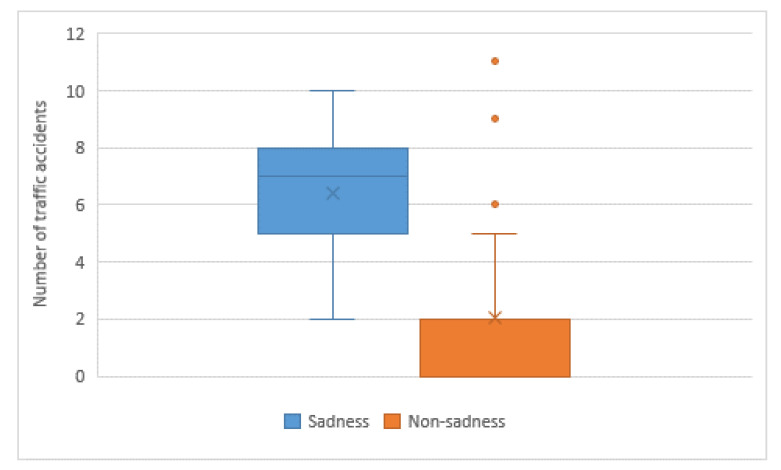
Number of traffic accidents grouped by sadness level.

Figure 4 captures the average number of traffic accidents. Drivers with sadness suffer traffic accidents more often than the group of drivers without sadness. The difference between the two groups is especially relevant, as the group with sadness is involved in four times as many accidents as the group of drivers without sadness. The result of Wilcoxon´s test is Z = −4.741, *p* < 0.05.

**Figure 5:**
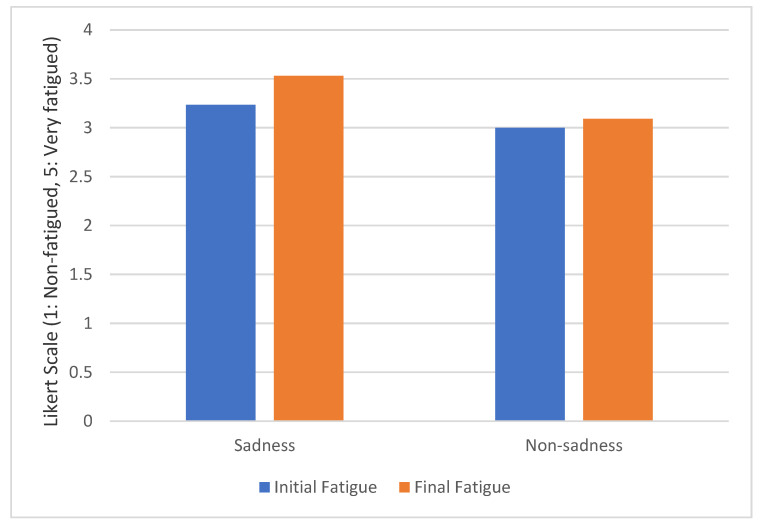
Fatigue evolution grouped by sadness level.

Figure 5 compares the level of fatigue before and after the driving test. Drivers suffering from sadness increased their fatigue level by 11.5% compared to their initial value. In the case of drivers without sadness, fatigue increased by 7.5%. However, we found no significant differences between both groups at the beginning and at the end of the experiment. On the one hand, the result of Wilcoxon´s test in the initial survey is Z = −0.361, *p* > 0.05. On the other hand, the result of Wilcoxon´s test in the post-experimental survey is Z = −1.472, *p* > 0.05.

### 4.3. Effects of Fatigue

In order to analyze this factor, we have divided the samples into two groups. The drivers indicated their initial fatigue level using a Likert scale with values between 1 and 5, where 1 means that they are very vigorous and 5 indicates that they are very tired. The non-fatigue group consists of 36 drivers. These drivers showed a tiredness level equal to or less than 3. The fatigue group is made up of 14 drivers. These indicated in the initial survey that their fatigue level was equal to or higher than 4.

Table 10 reveals the average stress level during the test grouped by the initial fatigue level. The results indicate that tired drivers suffer more stress while driving than the other drivers. The variable pNN50 is eight times lower in the group of drivers who are tired, and the LF/HF ratio and SCR amplitude are twice as high. Low values of pNN50 and high values of LF/HF ratio and SCR amplitude are correlated with high stress. In all variables, the differences are significant. The result of Wilcoxon´s test is Z = −4.905, *p* < 0.05 for pNN50, Z = −4.127, *p* < 0.05 for LF/HF and Z= −3.297 for SCR.

Driving behavior is also affected by this state. Table 11 shows the use of the accelerator and brake. Acceleration time and braking time is higher for tired drivers than for rested drivers. The differences are significant. The result of the Student’s test is t(48) = 2.905, *p* < 0.05 for acceleration time and t(48) = 3.754 *p* < 0.05 for braking time. This means that the drivers are continuously making speed corrections and increasing fuel consumption. No significant differences have been found in the case of abrupt maneuvers, although both average and median values are higher for tired drivers.

**Figure 6:**
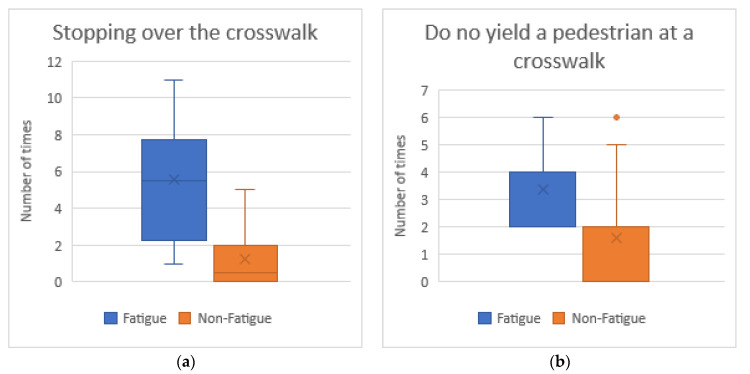
Average number of traffic rules broken grouped by tiredness level: (**a**) Stopping over the crosswalk; and (**b**) Do not yield to a pedestrian at a crosswalk.

Figure 6 captures the number of broken driving rules in which there are significant differences between fatigued and non-fatigued drivers. The result of Wilcoxon´s test is Z = −4.402, *p* < 0.05 for “Stopping over the crosswalk” and Z = −3.459, *p* < 0.05 for “Do not yield to a pedestrian at a crosswalk”. Tired drivers stop over the crosswalk 4.5 more times more than the rest of the drivers. Furthermore, they did not yield to a pedestrian at a crosswalk two times more. This could increase the likelihood of running over a pedestrian.

**Figure 7:**
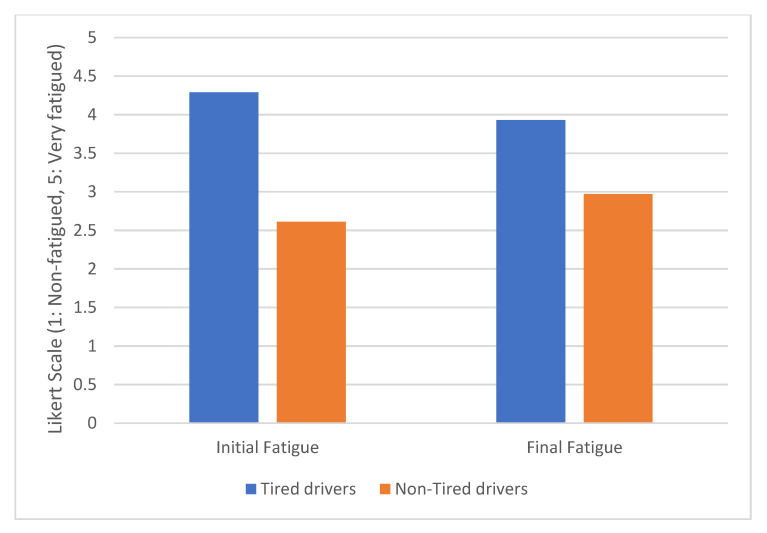
Fatigue evolution.

Figure 7 compares the difference between initial and final fatigue for the two groups of drivers. On the one hand, there is a significant increase in the fatigue level of the non-tired drivers. Tiredness increased by 13.79% after completing the driving test. On the other hand, in the case of tired drivers, the average fatigue value decreases by 8.39%. This could be because for some participants, the driving test is like a leisure activity. Despite this, the level of fatigue manifested by the drivers who were initially tired remains significantly higher than that of the drivers who initially did not feel tired. The result of Wilcoxon´s test is Z = −3.105, *p* < 0.05.

### 4.4. Effects of CO_2_ Concentration

In order to analyze this factor, we have divided the samples into two groups. One group consists of 29 drivers who drove with an average CO_2_ value of less than 1400 ppm. We have chosen this threshold because it has been shown in many articles [11] that differences in cognitive performance appear above this value. The average value of CO_2_ concentration of this group was 319.67 ppm (max: 562.55 ppm, min: 149.8 ppm, std. dev: 119.17 ppm). The second group is made up of 21 drivers who drove with an average CO_2_ value equal to or higher than 1400 ppm. The average value of CO_2_ concentration of this group was 1572.96 ppm (max: 1734.56 ppm, min: 1434.81 ppm, std. dev: 107.43 ppm). The average temperature value during all the tests was 25.27 °C (maximum = 26.71 °C, minimum = 24.12 °C, standard deviation = 0.63 °C) and the average humidity was 50.64% (maximum = 58.13%, minimum = 48.35%, standard deviation = 3.11%).

Table 12 captures the value of the variables associated with stress. The difference between groups is not significant. The result of Wilcoxon’s test is Z = −0.147, *p* > 0.05 for pNN50, Z = −0.88, *p* > 0.05 for LF/HF and Z = −0.364, *p* > 0.05 for SCR amplitude.

Table 13 shows driving behavior. The results indicate that the driver brakes more frequently when the passenger compartment has a high concentration of CO_2_. The difference is significant between the two groups (high and low CO_2_ level). The result of Wilcoxon´s test was Z = −3.843, *p* < 0.05 for braking time. A high CO_2_ concentration causes drowsiness and a lack of concentration. The participant’s cognitive capacity is reduced and he or she responds more slowly to events that happen on the road.

**Figure 8:**
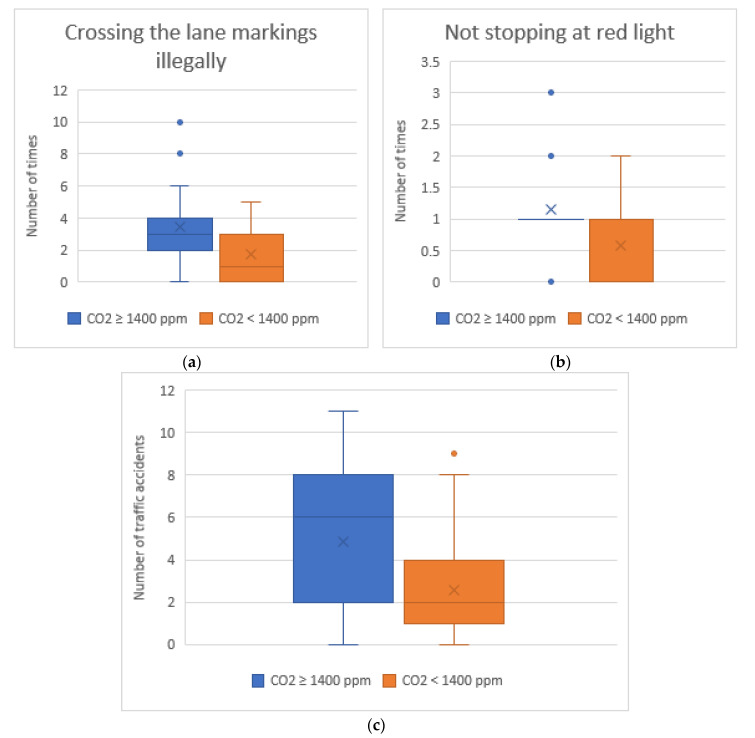
(**a**) Number of times drivers cross the lane markings illegally; (**b**) number of times that drivers do not stop at a red light; and (**c**) number of traffic accidents.

Consequently, as we can see in Figure 8, the driver violates more traffic regulations and is involved in a higher number of traffic accidents. We found significant differences in “Crossing the lane markings illegally” (Z = −2.478, *p* < 0.05), “Not stopping at a red light” (Z = −2.752, *p* < 0.05) and “Traffic accidents” (Z = −2.105, *p* < 0.05). Figure 8 captures the traffic rules broken and traffic accidents grouped by CO_2_ level. We can see how drivers who are exposed to high concentrations of CO_2_ invade the opposite lane 95% more than the rest of the drivers. Moreover, they respect traffic lights less. The group of drivers who drive with a high concentration of CO_2_ ignored the red lights 1.14 times on average, while the drivers who drive with a low CO_2_ level passed red lights 0.59 times. Frequent decelerations along with non-compliance with traffic regulations result in a sharp increase in the number of accidents of the group with the high CO_2_ concentration. These drivers suffer 1.87 times more accidents than the rest of the drivers.

**Figure 9:**
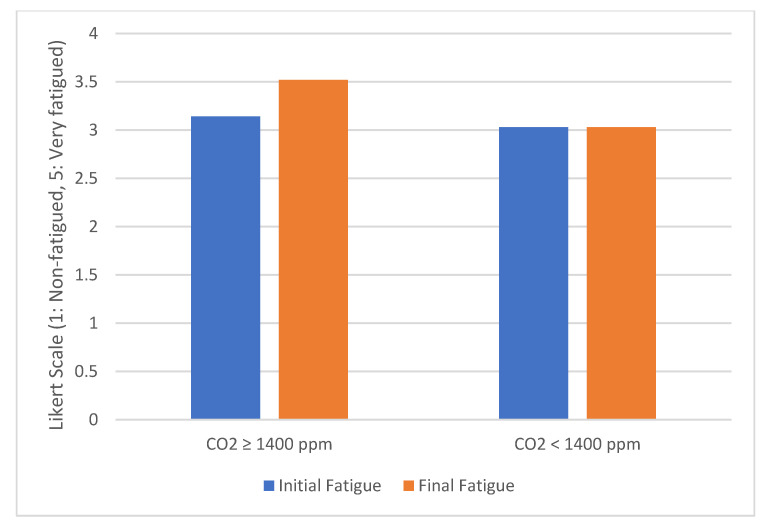
Evolution of fatigue grouped by CO_2_ level.

Figure 9 captures the initial and final level of fatigue for each group of drivers. The results show that when the CO_2_ concentration is high, the fatigue level increases by 12% compared to the initial value, while when the CO_2_ level is low, the level of fatigue does not change.

### 4.5. Effects of Music Tempo

As in the previous analyses, the driving samples were divided into two groups. One group is made up of 23 drivers who listened to slow tempo music. The other group consists of 27 participants, but in this case the music was fast tempo music.

Table 14 captures the value of the variables related to stress. In the case of drivers listening to fast-paced music, pnn50 and LF/HF ratio are higher than drivers listening to slow music, although the differences are not significant. The result of Wilcoxon’s test is Z = −0.049, *p* > 0.05 for pNN50, Z = −0.457, *p* > 0.05 for LF/HF and Z = −0.886, *p* > 0.05 for SCR amplitude.

**Figure 10:**
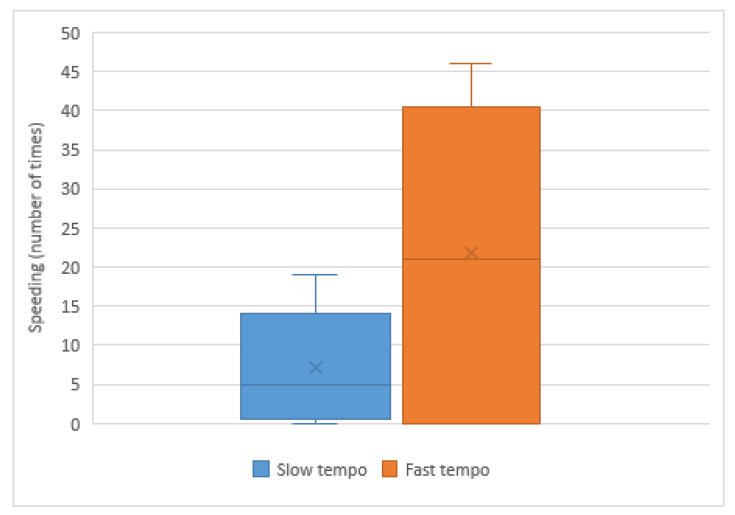
Number of times the driver exceeds the speed limit.

Table 15 captures driving behavior grouped by music tempo. We observed that the average values of the four variables analyzed are higher in the case of drivers who listen to fast-paced music than participants who listen to slow-paced music. This means that drivers with fast-paced music show a more aggressive driving style, although we have only found significant differences in acceleration time. The result of the Student’s test is t(48) = −2.891, *p* < 0.05. Likewise, we have found significant differences in the violation of speed limits, as can be seen in Figure 10. The result of Wilcoxon´s test is Z = −1.980, *p* < 0.05. As future work, we want to conduct more experiments to verify whether the differences between the driving behavior variables are significant if the number of participants is increased.

**Figure 11:**
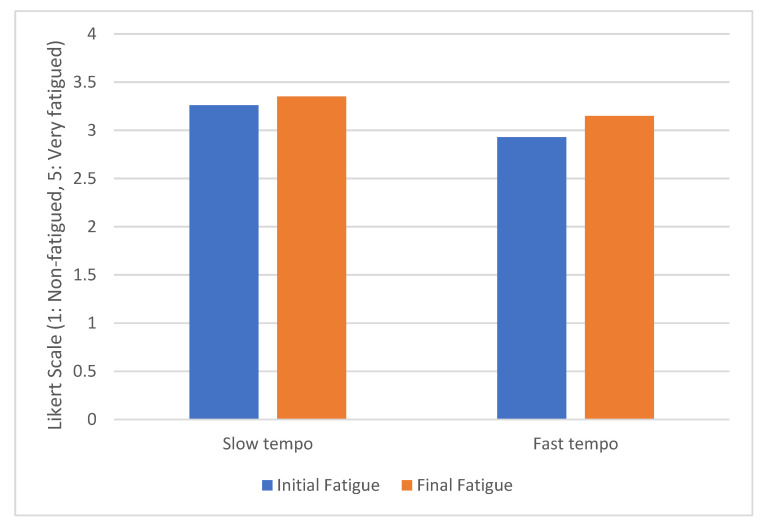
Fatigue evolution grouped by music tempo.

Figure 11 shows the level of initial and final fatigue for the two groups of drivers using a Likert scale, where 1 means no fatigue and 5 a lot of fatigue. The level of fatigue only increased by 2.8% for drivers who listened to music at a slow pace. In contrast, drivers who listened to fast-paced music suffered a significant increase in the level of fatigue (by 7.5%). These results are consistent with those obtained by [95]. In this study, fast music deteriorated the level of fatigue.

### 4.6. Multivariate Analysis

Linear ANOVA models have been calculated for each of the factors analyzed: initial stress, sadness, initial fatigue, CO_2_ concentration and music tempo. In all models, the *p* value is less than 0.05. Therefore, we can state that the independent variables reliably predict the dependent variable. Table 16 shows the models with an adjusted R-squared (R^2^) higher than 55%. Adjusted R-squared is a statistic that gives information about the goodness of fit of a model. R-squared is defined as the fraction of the variance in the dependent variable that is explained by the model. The adjusted R-squared is a modified version of R-squared that has been adjusted for the number of predictors in the model. The higher the adjusted R-squared value, the more the model fits the real data. In Table 16, the labels labeled as “COEFFICIENT” are the values for the regression equation for predicting the dependent variable from the independent variables. Finally, the *p* value is a probability. It gauges the likelihood that the coefficient is not significant, so smaller is better. In our case, we consider that there is significance when the value is less than 0.05.

We can see that the best model is obtained in “Speeding”, where the adjusted R^2^ is higher than 70%. In view of the coefficient’s values and the *p* value, we can state that the initial stress level as well as the initial fatigue and fast-paced music significantly increase the number of times the speed limits are surpassed. We can also point out that both sadness and a high concentration of CO_2_ do not seem to influence speeding. In these two independent variables, the *p* values are higher than 0.05.

In the LF/HF ratio, we found that the initial stress level along with fatigue contributes to the occurrence of stress during driving. It is important to highlight the strong relationship between initial fatigue and the possibility of stress while driving, where the coefficient value is 4.152. In the case of the other two variables related to driving stress (pNN50 and SCR amplitude), the same thing happens, but we have not included them in the table because the adjusted R-squared value is lower than 50%. Finally, we can observe that when the driver suffers sadness, the value of the LF/HF ratio decreases, meaning less stress in driving. The *p* value for sadness is lower than 0.05 and the coefficient is −1.405. This could be explained because the drivers are focused on their own problems. Extremely low driving stress is also not good for safety because it could cause drowsiness [5].

The results of the “Harsh braking” variable are very similar to the “LF/HF” variable. However, the *p* value of sadness is higher than 0.05. Therefore, in this context, it does not significantly affect the model. The driver who is initially tired or stressed does not react early enough to road events, forcing aggressive maneuvers and increasing driving stress.

Regarding sudden accelerations, they characterize an aggressive driving style which appears especially when the driver is stressed. The coefficient value is 6.936 and the *p* value is lower than 0.05. Sadness is also an emotion that contributes. The coefficient value is 2.315 and the *p* value is lower than 0.05. People with sadness often adopt an aggressive driving style and a certain degree of passiveness that causes increased fuel consumption and can annoy other drivers [22]. On the contrary, a high concentration of CO_2_ decreases harsh accelerations. The coefficient value is −2.074 and the *p* value is lower than 0.05. This could be due to the possible appearance of drowsiness [96].

## 5. Discussion and Limitations of Our Experiment

In our experiment, the initial level of stress and fatigue has a strong impact on driving behavior and driving stress. The relationship between stress and road safety has been verified by many authors [97,98]. Several studies have corroborated that a high level of stress increases errors and traffic violations. In [46], the authors conducted a study involving 2806 drivers using the driver behavior questionnaire (DBQ) and the driver behavior inventory (DBI). The DBI assesses dimensions of driver stress, whereas the DBQ is concerned with assessing the relative frequencies with which drivers engage in different types of aberrant driving behavior. They found a strong correlation between an aggressive driving style and high levels of stress. They also observed that when the stress is high, drivers make more mistakes, although in this case, the dislike of driving also seems to play a role. This is consistent with our findings that stressed drivers accelerate and brake more often than non-stressed drivers. Furthermore, harsh accelerations are six times higher than the values obtained by non-stressed drivers. In the case of harsh braking, the values are twice as high as those obtained by non-stressed drivers. Harsh accelerations and harsh braking are indicative of an aggressive driving style. The main difference between our analysis and the previous literature is that we have monitored the driver’s state and driving behavior. Most of the proposals are based on self-reports of drivers or traffic accident databases provided by the government [99]. The problem with self-reports is that they depend on the drivers’ perception, which could be wrong. In [99], the authors found that drivers with high confinement had a low risk perception and reported driving errors incorrectly.

Regarding the sadness factor, we observe that it is mainly characterized by a very significant increase in the number of traffic accidents. This emotion also contributes significantly to the increase in sudden decelerations. Attentional self-focus and repetitive negative thoughts are two main elements in sadness [100,101]. These elements affect information processing and attention [102]. In [103], the authors observed that sadness-induced drivers made more errors in target location. This could explain why, in our experiment, drivers with sadness suffered more traffic accidents than drivers who do not feel this emotion. On the other hand, we also found in our driving test that drivers with sadness did not manifest more stress than other drivers. In [22], the researchers conducted a simulated driving experiment with two induced affective states to examine how sadness and anger differently influence driving-related risk perception, driving performance and perceived workload. The results they obtained showed that sad drivers make more driving errors, but do not perceive a higher workload than drivers with an emotionally neutral state. This could explain why we have not found significant differences in driving stress.

In the literature, many researchers focus on analyzing how fatigue that increases during driving affects driving performance and road safety [104]. These studies point out that fatigue is a very important factor that causes a lack of hazard perception [105]. This may lead to driving accidents [106]. In this regard, the European Union has a regulation that sets the maximum driving time for professional drivers [107]. The relationship between driver fatigue and hours of service regulations is a challenge [108]. Some authors have found that driving time is a significant predictor of accident risk [109]. In other studies, there is no evidence of a time-on-task effect [110]. This could be due to the repercussion of the driver’s initial fatigue level. In our study, we have observed that initial fatigue significantly influences driving behavior and driving stress. We have also observed a non-compliance with traffic regulations that require high attention from the subject such as “yield to a pedestrian at a crosswalk”. This demonstrates the need to not only monitor fatigue during driving, but also to do so beforehand in order to ensure driving safety.

Traditionally, the CO_2_ concentration inside the vehicle cabin was not considered dangerous because of its low level. However, several recent studies have shown that the concentration of CO_2_ can be quite high depending on the number of vehicle occupants, speed and the environment [111]. In addition, cognitive impairment has also been observed with low or moderate CO_2_ concentrations with short exposure times [112]. In [113], the authors observed that the mental task required more effort from the subjects when the CO_2_ concentration in the air reached 3000 ppm. In [12], the researchers concluded that decision-making performance decreased when participants were exposed to CO_2_ concentrations between 1000 and 2500 ppm. This is in line with what was observed in our study. The worsening of decision making when the CO_2_ concentration is high causes the number of traffic accidents to increase. A high CO_2_ concentration also causes fatigue and drowsiness in drivers, reducing reaction time [114]. As a consequence, we observed in our study an increase in the frequency and intensity of decelerations. Finally, the combination of high initial stress with fast-paced music causes, in our experiment, a significant increase in the number of times the maximum allowed speed is exceeded. There are many marketing studies where fast music is used to encourage customers to purchase [115,116]. In the field of driving, many researchers have observed a similar behavior. In [90], the authors concluded that listening to fast music in the background affects non-compliance with traffic rules such as speeding.

As a limitation in our study, we did not take into account variables such as personality, gender, socio-educational level or the driver’s history (fines and traffic accidents). In [117], the researchers conducted a study with 41 drivers using a driving simulator, where they observed that these variables affect driving behavior, especially when drivers are tired. These factors were not included in the survey in order not to extend our experiment and discourage participants. In most of the papers, the subjects only had to fill out surveys and did not drive. Another limitation is in the evaluation of the music factor. We have only analyzed the tempo. The subject could freely adjust the volume of the music and the playlist was the same for all participants. We have not considered other elements that can influence driving behavior such as gender or music familiarity [118].

## 6. Conclusions

In this work, we have analyzed how the mental state of the driver and the interior state of the vehicle affects driving and its relation to compliance with traffic regulations and accidents.

Among the factors analyzed, the negative influence of stress stands out. On the one hand, stress is strongly related to an aggressive driving style with sudden accelerations and decelerations. This behavior means that the rest of the road users are not able to predict their actions, increasing the probability of traffic accidents. In the driving tests, these drivers did not often respect the speed limits, they overtook other vehicles in areas where this action should not be performed and did not stop at the crosswalks. On the other hand, the driving style associated with this state increases fuel consumption. As the driver drives at an inappropriate speed, the brakes are used more, and the driver does not take advantage of the energy generated by burning the fuel.

Sadness also influences driving behavior. This emotion in combination with stress and listening to fast music increases the number of harsh accelerations, causing problems for both safety and the environment. Drivers suffering from sadness are frequently involved in traffic accidents because they are thinking about their own problems and do not focus on paying attention to the road.

Tiredness is another analyzed factor that has negative consequences. We have observed that tired drivers suffer more stress while driving than non-tired drivers. Tiredness increases response times, and as a result, drivers accelerate and brake more frequently. This could cause a traffic accident because the driver of the vehicle behind only has a short time to react.

Furthermore, we have observed that drivers who listen to music with a fast tempo drive at high speeds, not respecting the limits indicated on the traffic signs. High-speed driving demands more cognitive ability. If the demand is prolonged, it causes an increase in the level of fatigue.

Regarding the interior state of the vehicle, the results obtained when analyzing the data of drivers who were exposed to high concentrations of CO_2_ are very similar to those of drivers who were tired. A high concentration of CO_2_ causes fatigue and headache, reducing the concentration of the driver on the road. Finally, we want to highlight that we have observed that some drivers who liked video games and were very stressed or tired improved their initial state when doing the driving test. This result could be very useful for developing driving assistants.

In conclusion, this work shows that the driver’s behavior not only depends on the driving conditions, but that it is also influenced by the driver’s state. Factors such as stress or fatigue can intensify while driving, but the initial values before driving are also very relevant and strongly related to more erratic and dangerous driving. Researchers working on the design of driving assistants could explore whether issuing lifestyle advice improves driving safety and driving efficiency.

As future work, we would like to evaluate how the personality of the driver impacts driving. This, combined with the results obtained in this work, would allow us to develop an advanced driving assistant (ADAS) that fits with the driver profile. An ADAS could intelligently influence the driver’s emotions.

## Figures and Tables

**Table 1:** Polar H10 specifications. Data from [64].

**Battery Type**	CR 2025
**Battery sealing ring**	O-ring 20.0 × 0.90 Material Silicone
**Battery lifetime**	400 h
**Sampling rate**	1 Hz
**Operating temperature**	−10 °C to +50 °C/14 °F to 122 °F
**Connector material**	ABS, ABS + GF, PC, Stainless steel
**Strap material**	38% Polyamide, 29% Polyurethane, 20%

**Table 2:** Empatica E4 specifications. Data from [65].

**PPG sensor**	Sampling frequency 64 Hz LEDs: Green (2 LEDs), Red (2 LEDs) Photodiodes: 2 units, total 15.5 mm^2^ sensitive area Sensor output resolution 0.9 nW/Digit
**EDA sensor**	Sampling frequency: 4 Hz Resolution: 1 digit ~900 pSiemens Range: 0.01 μSiemens–100 μSiemens
**Infrared thermopile**	Sampling frequency: 4 Hz Range: −40…115 °C Resolution: 0.02 °C Accuracy ±0.2 °C within 36–39 °C
**3-Axis accelerometer**	Sampling frequency: 32 Hz Range ±2 g
**E4 operating range**	Relative Humidity 60 ± 25% H.R.
**Water resistance**	IP 22
**Memory**	Device storage capacity exceeds 60 recording hours.
**Connectivity**	Bluetooth LE Operating range: 10 m
**Size**	44 × 30 × 16 mm

**Table 3:** Specifications of the Netatmo Indoor Air Quality Monitor. Data from [81].

**Temperature**	Range	0 °C to 50 °C
	Accuracy	±0.3 °C
**Humidity**	Range	0 to 100%
	Accuracy	±3%
**CO_2_**	Range	0 to 5000 ppm
	Accuracy	±50 ppm (from 0 to 1000 ppm) or ±5% (from 1000 to 5000 ppm)
**Sound meter**	Ranges from: 35 to 120 dB	
**Records frequency**	Every 5 min	
**Connectivity specifications**	Wi-Fi 802.11 b/g/n compatible (2.4 GHz) Supported security: Open/WEP/WPA/WPA2-personal (TKIP and AES)	
**Size**	45 × 45 × 155 mm	

**Table 4:** Specifications of the PC on which the driving simulator is run.

**Model**	Alienware Area-51 R4
**Processor**	Intel Core i7-7800X
**Chipset**	Intel X299 PCH
**Memory**	16 GB DDR4 2666 MHz
**GPU**	2 X Geforce 1080 TI SLI
**Storage**	128 GB SanDisk M.2 SSD

**Table 5:** Specifications of the G29. Data from [84].

**Wheel**	Rotation: 900 degrees lock-to-lock Hall-effect steering sensor Dual-Motor Force Feedback Overheat safeguard
**Pedals**	Nonlinear brake pedal Patented carpet grip system Textured heel grip Self-calibrating
**Size**	Wheel: 270 × 260 × 278 mm Pedals: 167 × 428.5 × 311 mm
**Connection**	USB 2.0
**Compatible OS**	Windows 10, 8.1 Windows 8 or Windows 7 macOS 10.10 Playstation 4 or Playstation 3

**Table 6:** Heart rate variability and skin conductivity during the driving test grouped by initial stress level.

		Stressed	Non-Stressed	*p* Value
**pNN50**	Average Value	5.08%	17.95%	0.002
	Median Value	2.90%	10.88%	
	Std. Deviation	7.03%	19.96%	
	P25	1.07%	3.20%	
	P75	5.52%	23.25%	
**LF/HF**	Average Value	6.83	3.61	<0.001
	Median Value	6.28	2.83	
	Std. Deviation	2.64	2.80	
	P25	4.95	1.22	
	P75	8.86	5.93	
**SCR Amplitude**	Average Value	0.55 µS	0.25 µS	<0.001
	Median Value	0.48 µS	0.11 µS	
	Std. Deviation	0.35 µS	0.31 µS	
	P25	0.33 µS	0.08 µS	
	P75	0.71 µS	0.26 µS	

**Table 7:** Driving behavior grouped by initial stress level.

		Stressed	Non-Stressed	*p* Value
**Harsh braking**	Average Value	24.43%	9.12%	<0.001
	Median Value	25.25%	7.47%	
	Std. Deviation	6.38%	6.20%	
	P25	19.36%	4.95%	
	P75	28.80%	10.10%	
**Braking time**	Average Value	24.55%	17.86%	0.015
	Median Value	24.05%	17.11%	
	Std. Deviation	6.77%	9.27%	
	P25	21.94%	9.05%	
	P75	29.26%	25.35%	
**Harsh Acceleration**	Average Value	8.16%	1.37%	<0.001
	Median Value	6.48%	0.48%	
	Std. Deviation	4.83%	1.87%	
	P25	6.14%	0.15%	
	P75	12.58%	1.65%	
**Acceleration time**	Average Value	67.01%	61.018%	0.009
	Median Value	68.67%	61.34%	
	Std. Deviation	7.69%	7.40%	
	P25	60.43%	55.11%	
	P75	72.94%	66.02%	

**Table 8:** Heart rate variability and skin conductivity during the driving test grouped by sadness level.

		Sadness	Non-Sadness	*p* Value
**pNN50**	Average Value	10.90%	13.39%	0.384
	Median Value	3.94%	6.66%	
	Std. Deviation	17.94%	16.72%	
	P25	1.12%	2.61%	
	P75	11.58%	17.08%	
**LF/HF**	Average Value	4.68	5.11	0.647
	Median Value	4.40	4.67	
	Std. Deviation	3.10	3.21	
	P25	2.10	2.65	
	P75	7.19	7.68	
**SCR Amplitude**	Average Value	0.45 µS	0.34 µS	0.682
	Median Value	0.26 µS	0.26 µS	
	Std. Deviation	0.41 µS	0.32 µS	
	P25	0.12 µS	0.10 µS	
	P75	0.89 µS	0.48 µS	

**Table 9:** Driving behavior grouped by sadness level.

		Sadness	Non-Sadness	*p* Value
**Harsh braking**	Average Value	16.36	15.23	0.757
	Median Value	16.69	14.39	
	Std. Deviation	11.03	9.34	
	P25	7.47	5.89	
	P75	26.69	24.03	
**Braking time**	Average Value	20.73	20.64	0.935
	Median Value	23.74	21.05	
	Std. Deviation	8.29	9.30	
	P25	14.47	11.45	
	P75	26.40	29.26	
**Harsh Acceleration**	Average Value	6.06	3.28	0.051
	Median Value	5.51	1.02	
	Std. Deviation	5.36	4.26	
	P25	1.09	0.33	
	P75	9.06	6.18	
**Acceleration time**	Average Value	64.55	63.16	0.565
	Median Value	61.04	63.15	
	Std. Deviation	8.35	7.90	
	P25	57.45	59.45	
	P75	70.91	68.67	

**Table 10:** Heart rate variability and skin conductivity during driving test grouped by tiredness level.

		Fatigue	Non-Fatigue	*p* Value
**pNN50**	Average Value	1.47%	16.85%	<0.001
	Median Value	1.10%	9.45%	
	Std. Deviation	1.45%	18.32%	
	P25	0.60%	3.89%	
	P75	2.15%	21.66%	
**LF/HF**	Average Value	7.96	3.80	<0.001
	Median Value	7.48	3.08	
	Std. Deviation	1.82	2.76	
	P25	6.83	1.77	
	P75	8.50	4.96	
**SCR Amplitude**	Average Value	0.62 µS	0.28 µS	0.001
	Median Value	0.61 µS	0.16 µS	
	Std. Deviation	0.35 µS	0.31 µS	
	P25	0.28 µS	0.08 µS	
	P75	1.00 µS	0.44 µS	

**Table 11:** Driving behavior grouped by level of tiredness.

		Fatigue	Non-Fatigue	*p* Value
**Harsh braking**	Average Value	16.16%	15.23%	0.757
	Median Value	16.69%	14.39%	
	Std. Deviation	11.03%	9.34%	
	P25	7.47%	5.89%	
	P75	26.69%	24.03%	
**Braking time**	Average Value	27.39%	18.06%	<0.001
	Median Value	26.50%	17.37%	
	Std. Deviation	5.33%	8.65%	
	P25	24.07%	10.09%	
	P75	29.51%	24.29%	
**Harsh Acceleration**	Average Value	5.74%	3.64%	0.166
	Median Value	5.83%	1.19%	
	Std. Deviation	5.24%	4.55%	
	P25	0.81%	0.36%	
	P75	6.85%	6.31%	
**Acceleration time**	Average Value	68.54%	61.72%	0.006
	Median Value	68.83%	60.96%	
	Std. Deviation	7.36%	7.48%	
	P25	62.79%	55.37%	
	P75	75.22%	67.93%	

**Table 12:** Heart rate variability and skin conductivity during driving test grouped by CO_2_ level.

		CO_2_ ≥ 1400 ppm	CO_2_ < 1400 ppm	*p* Value
**pNN50**	Average Value	15.21%	10.61%	0.891
	Median Value	3.94%	5.57%	
	Std. Deviation	20.11%	14.41%	
	P25	1.75%	2.61%	
	P75	28.31%	10.93%	
**LF/HF**	Average Value	5.01	4.94	0.938
	Median Value	4.67	3.53	
	Std. Deviation	3.32	3.08	
	P25	2.65	2.49	
	P75	6.76	7.28	
**SCR Amplitude**	Average Value	0.39 µS	0.37 µS	0.723
	Median Value	0.29 µS	0.20 µS	
	Std. Deviation	0.34 µS	0.37 µS	
	P25	15.21%	10.61%	
	P75	3.94%	5.57%	

**Table 13:** Driving behavior grouped by CO_2_ level.

		CO_2_ ≥ 1400 ppm	CO_2_ < 1400 ppm	*p* Value
**Harsh braking**	Average Value	18.66%	13.30%	0.070
	Median Value	17.45%	9.57%	
	Std. Deviation	10.25%	9.05%	
	P25	9.15%	5.80%	
	P75	26.69%	20.58%	
**Braking time**	Average Value	26.40%	16.52%	<0.001
	Median Value	27.47%	14.90%	
	Std. Deviation	7.69%	7.31%	
	P25	22.74%	9.87%	
	P75	31.24%	23.93%	
**Harsh Acceleration**	Average Value	3.85%	4.50%	0.930
	Median Value	4.24%	1.65%	
	Std. Deviation	3.83%	5.44%	
	P25	0.48%	0.33%	
	P75	6.54%	7.26%	
**Acceleration time**	Average Value	63.53%	63.70%	0.943
	Median Value	61.04%	63.15%	
	Std. Deviation	7.87%	8.22%	
	P25	59.22%	57.45%	
	P75	70.36%	69.43%	

**Table 14:** Heart rate variability and skin conductivity during the driving test grouped by music tempo.

		Slow Tempo	Fast Tempo	*p* Value
**pNN50**	Average Value	12.30%	12.76%	0.969
	Median Value	3.94%	7.10%	
	Std. Deviation	17.20%	17.15%	
	P25	2.41%	1.44%	
	P75	12.72%	16.80%	
**LF/HF**	Average Value	4.65	5.23	0.657
	Median Value	4.95	3.53	
	Std. Deviation	2.32	3.73	
	P25	3.01	1.95	
	P75	6.26	8.74	
**SCR Amplitude**	Average Value	0.40 µS	0.36 µS	0.381
	Median Value	0.35 µS	0.25 µS	
	Std. Deviation	0.33 µS	0.38 µS	
	P25	0.14 µS	0.08 µS	
	P75	0.44 µS	0.44 µS	

**Table 15:** Driving behavior grouped by music tempo.

		Slow Tempo	Fast Tempo	*p* Value
**Harsh braking**	Average Value	14.48%	16.45%	0.428
	Median Value	15.51%	12.99%	
	Std. Deviation	8.81%	10.72%	
	P25	6.03%	7.05%	
	P75	19.33%	26.57%	
**Braking time**	Average Value	20.04%	21.21%	0.649
	Median Value	21.94%	22.74%	
	Std. Deviation	9.38%	8.58%	
	P25	10.75%	14.68%	
	P75	26.50%	29.45%	
**Harsh Acceleration**	Average Value	3.56%	4.79%	0.876
	Median Value	1.65%	2.03%	
	Std. Deviation	4.02%	5.38%	
	P25	0.55%	0.20%	
	P75	5.37%	7.10%	
**Acceleration time**	Average Value	60.33%	66.44%	0.006
	Median Value	59.56%	68.05%	
	Std. Deviation	7.70%	7.24%	
	P25	57.04%	62.70%	
	P75	61.75%	70.86%	

**Table 16:** Results of multivariate analysis with high R^2^.

	Factor	Coefficient	*p* Value	R^2^
**LF/HF**	High Initial Stress	2.744	<0.001	56.69%
	Sadness	−1.405	0.032	
	Tiredness	4.152	<0.001	
	High CO_2_ Concentration	−0.672	0.273	
	Fast Music	1.138	0.062	
**HARSH ACCELERATION**	High Initial Stress	6.936	<0.001	57.56%
	Sadness	2.315	0.020	
	Tiredness	0.586	0.574	
	High CO_2_ Concentration	−2.074	0.028	
	Fast Music	1.469	0.108	
**HARSH BRAKING**	High Initial Stress	14.234	<0.001	63.79%
	Sadness	−1.215	0.506	
	Tiredness	4.509	0.026	
	High CO_2_ Concentration	2.567	0.145	
	Fast Music	2.978	0.086	
**SPEEDING**	High Initial Stress	23.641	<0.001	71.72%
	Sadness	−3.884	0.162	
	Tiredness	6.209	0.042	
	High CO_2_ Concentration	−0.538	0.838	
	Fast Music	16.003	<0.001	
